# Early response and outcomes of bone marrow to chemotherapy in T-Cell Acute Lymphoblastic Leukemia

**DOI:** 10.12669/pjms.40.5.7584

**Published:** 2024

**Authors:** May AlMoshary, Shatha Mahmoud Altahan, Aziza Fayed Alswayyed

**Affiliations:** 1 May AlMoshary, Assistant Professor, Basic Science Department, College of Medicine, Princess Nourah bint Abdulrahman University, Riyadh, Saudi Arabia; 2 Shatha Mahmoud Altahan, Hematology Unit, Department of Pathology and Clinical Laboratory Medicine, Administration, King Fahad Medical City, Saudi Arabia; 3 Aziza Fayed Alswayye, Hematology Unit, Department of Pathology and Clinical Laboratory Medicine, Administration, King Fahad Medical City, Saudi Arabia

**Keywords:** T-ALL, cancer, Leukemia, Bone marrow, Outcomes, Relapse

## Abstract

**Objectives:**

To evaluate the outcomes (relapse and mortality rate) and response of the bone marrow in early stages after combination chemotherapy in patients with T-cell Acute Lymphoblastic Leukemia (T-ALL)

**Methods:**

A descriptive cross-sectional study was conducted at King Fahad Medical City, from January 2021 to December 2022, to evaluate bone marrow findings at the time of diagnosis and post-chemotherapy in 26 patients diagnosed with T-ALL. The study included all patients diagnosed with T-ALL of any age group during the study period. The patients’ bone marrows were examined at 0 days of treatment (diagnosis work-up), followed by examination at day 15 post induction therapy, and day 30 after treatment.

**Results:**

In this study, 26 cases of T-lymphoblastic leukemia were analyzed. The mean age at diagnosis was 15.69±14.28 years, and eight cases had central nervous system involvement. The majority of cases (88.5%) were positive for Cytoplasmic-CD3 and CD7. Positive findings by fluorescence in situ hybridization (FISH) were: T cell receptor (TCR) α/δ in 6 (23.1%) of the patients, CDNK2A/CEP9 in five (19.2%), and TRCB in one (3.8%). Examination of the bone marrow on day 15 revealed a decrease in blasts to ≤1% in nine patients, and to ≤1% in 19 patients on day 30 post-therapy. Relapse was recorded in five (19.23%) patients. Three (11.53%) patients did not survive during treatment, of which two were <10 years old. The relapse rate for T-ALL was 19.23%, with an overall survival rate of about 64%. The overall mortality rate was 11.53%.

**Conclusion:**

The relapse rate for T-ALL in our study was approximately 19%, but the mortality rate was 11.5%. A substantial decrease in blast percentages was observed, suggesting a favorable initial reaction of the bone marrow to the combined chemotherapy. This suggests that the use of aggressive and more effective chemotherapy has led to better outcomes.

## INTRODUCTION

Acute Lymphoblastic Leukemia (ALL) is a type of hematologic cancer characterized by the development of immature lymphoid cells (blasts) in the bone marrow, peripheral blood, central nervous system (CNS), testicles, and other organs.^1^ It is the most common form of childhood cancer, accounting for 75-80% of pediatric acute leukemias.^2^ ALL can originate from either B- or T-cells, with B-cell ALL accounting for 80-85% and T-cell ALL accounting for 20-25%.^3^

T-cell acute lymphoblastic leukemia (T-ALL) is distinct from B-cell acute lymphoblastic leukemia (B-ALL) in terms of disease response patterns. Although similar regimens are used to treat T-ALL and B-ALL, there have been reported differences in responsiveness to various aspects of therapy.^4^ T-ALL has a high relapse rate after achieving remission with chemotherapy, which contributes to the inferior overall survival (OS) rate of 50-60% in adult patients.^5^ However, with the implementation of risk-adapted therapy and improved supportive care, the survival rate of T-ALL in children has increased from 57% to 92%.^6^,^7^ However, a lower survival rate is reported in developing countries, ranging from 30-70%.^8^

Relapse occurs in 20% of children and 40-50% of adults diagnosed with T-ALL.^9^ This has contributed to the poor outcome of this disease.^10^ In addition to other adverse factors, such as the higher frequency of high-risk genetic abnormalities and lower tolerance to intensive chemotherapy in older patients.^3^ T-ALL is a heterogeneous malignancy and adverse prognostic factors, such as complex karyotype, lack of mutation in either NOTCH1 or FBXW7 genes, del(17p), and ETP (early T-cell precursor) phenotype, can significantly increase the risk of relapse in T-ALL patients.^6^,^11^ The outcomes of adult ALL therapies are unsatisfactory.^12^

The low incidence of T-cell ALL compared to B-cell ALL has made it difficult for researchers to identify the clinical and biological factors that determine the outcome of T-ALL.^13^ Limited progress has been made in the treatment of T-ALL compared to B-ALL, therefore, novel and less expensive therapies need to be investigated to improve the outcomes in patients with T-ALL. In this study, we evaluated the response of the bone marrow at early stages after combination therapy of Vincristine and other drugs in T-ALL patients. Although there is some local published data about ALL in general, literature about T-ALL is still scarce compared to other leukemias. Publishing this paper will help to improve our understanding of this rare entity and solidify our knowledge about the disease characteristics of our patient population.

## METHODS

This was a descriptive cross-sectional study, in which the bone marrow responses of 26 patients who were diagnosed with T-ALL at King Fahad Medical City were investigated from January 2021 to December 2022. Patients received combination chemotherapy that included vincristine. The patients’ bones marrows were examined at zero days of treatment (diagnosis work-up), with included flow cytometry immunophenotyping, Fluorescence In Situ Hybridization (FISH), and molecular testing on some patients, followed by examination at day 15 post induction therapy that included bone marrow morphological assessment (cellularity and blast level) and at day 30 after treatment that included bone marrow assessment, flow cytometry and FISH markers for patients who had cytogenetic abnormalities.. All patients who were diagnosed with T-ALL of any age group were included in the study.

### Inclusion & Exclusion Criteria

Patients with acute leukemias other than T-ALL were not included in the study. Patients who did not complete the aforementioned bone marrow assessment steps at our center (e.g., continued therapy elsewhere) were also excluded.

### Statistical Analysis

Data analysis was done by Statistical Package for the Social Sciences (SPSS) program version 26. Descriptive statistics was used to calculate Mean±standard deviation for all numerical variables while frequencies & percentages was calculated for categorical variables. Kaplan Meier survival curve analysis method was used for the comparison of five years OS rates.

### Ethical Approval

It was obtained from King Fahad Medical city (OHRP/NIH, USA: IRB00010471 Date: March 09, 2022)

## RESULTS

In this study 26 cases with T-lymphoblastic leukemia were included in which majority were males 20 (76.9%). The mean age of diagnosis was 15.69±4.28 years. Eight cases had central nervous system involvement. The complete blood count (CBC) and patient demographics at diagnosis are given in Table-I.

**Table-I:** Demographic characteristics of the study participants (n=26).

Variables	Frequency (%)
***Gender***	
Male	20(76.9%)
Female	6(23.1%)
Age at diagnosis (mean/SD)	15.69±14.28
***Age groups***	
1-10 years	13(50%)
>10 years	13(50%)
CNS involvement	8(30.76%)
***Peripheral smear on diagnosis***	
Hemoglobin mg/dl	9.86±2.20
White blood cells x 10^9 (mean/SD)^	77.75±102.09
White blood cells <50 x 10^9^	17(65.4%)
White blood cells >50 x 10^9^	9(34.6%)
Platelets	95.04±82.136
Blast %	61±28.4

Immunophenotyping of all 26 cases was done at the time of diagnosis. Most of the cases 23(88.5%) were positive for CYTO-CD3 and CD7 each, followed by CD5, in which 21(80.8%) were positive. details of the immunophenotyping are given in Table-II.

**Table-II:** Diagnostic Immunophenotyping done by multi parameter flow cytometry (n=26).

Immunophenotyping	Positive	Negative	Partial positive*
CYTO-CD3	23(88.5%)	3(11.5%)	00
TdT	11(42.5%)	12(46.15%)	3(11.5%)
CD99	20(76.9%)	5(19.23%)	1(3.8)
CD1a	5(19.2%)	20(76.92%)	1(3.8%)
CD34	5(19.2%)	18(69.23%)	3(11.5%)
HLA-DR	4(15.4%)	20(76.92%)	2(7.7%)
S-CD3	2(7.7%)	23(88.5%)	1(3.8%)
CD4	12(46.2%)	14(53.84%)	00
CD8	6(23.1%)	17(65.38%)	3(11.5%)
CD7	23(88.5%)	3(11.5%)	00
CD2	20(76.9%)	4(15.4%)	2(7.7%)
CD5	21(80.8%)	3(11.5%)	2(7.7%)

* For a marker to be considered partially positive, 20% of the population in question should express that marker.

Routine cytogenetic studies were performed for all 26 cases. Specific common translocations seen in T-ALL were tested using fluorescence in situ hybridization (FISH). TCR A/D was positive in six (23.1%) of the patients, CDNK2A/CEP9 was positive in five (19.2%), TRCB was positive in only one (3.8%), TLX3 was positive in three (11.5%), and TLX1 was negative in all patients. The bone marrow examination on day 15 (n=26) revealed a decrease in blasts to ≤1% in nine patients, and to ≤1% in 19 patients on day 30 post-therapy Table-III.

**Table-III:** Early analysis of bone marrow on day 0, 15, and 30, of patients with outcome and relapse. (n=26).

Patient	Gender	Age	Day 0			Day 15			Day 30			Outcome	Relapse

			Blasts % by morphology*	Cellularity	Blasts % by FCM^$^	Blast % by morphology	Cellularity	Blasts % by FCM	Blasts % by morphology	Cellularity	Blasts % by FCM	Alive/dead	Yes/no
1	M	10	32%	Na	70%	<1%	80%	<0.01%	<1%	30%	<0.01%	Alive	No
2	F	6	35%	Na	80%	1%	40%	0.10%	<1%	20%	0.10%	Alive	No
3	M	11	1%	Na	20%	1%	40%	Na	<1%	40%	0.3%	Alive	No
4	M	17	90%	Na	90%	4%	60%	Na	<1%	30%	<0.01%	Alive	No
5	M	14	20%	Na	78%	2%	Na	2.80%	4%	20%	0.40%	Alive	Yes
6	M	9	20%	Na	35%	Na	35%	Na	Na	Na	Na	Deceased	No
7	M	34	84%	Na	80%	1%	40%	Na	1%	10%	<1%	Alive	No
8	F	10	95%	Na	75%	18%	40%	16%	<1%	5%	<1%	Alive	No
9	M	6	50%	Na	75%	25%	50%	Na	<1%	50%	<1%	Alive	No
10	M	9	15%	Na	25%	5%	40%	5.50%	<1%	20%	1%	Alive	No
11	F	21	95%	Na	70%	<1%	60%	<1%	<1%	30%	1%	Alive	Yes
12	M	10	92%	Na	NA	NA	NA	NA	NA	NA	NA	Deceased	No
13	M	10	30%	Na	50%	<1%	20%	Na	<1%	50%	<1%	Alive	No
14	F	3	78%	Na	74%	<1%	40%	Na	<1%	40%	3.5%	Alive	No
15	M	7	64%	Na	68%	<1%	40%	NA	NA	NA	NA	Deceased	Yes
16	M	70	87%	Na	75%	60%	90%	74%	40%	30%	5%	Alive	No
17	M	13	70%	Na	65%	65%	40%	Na	<1%	90%	33%	Alive	Yes
18	M	13	50%	Na	50%	2%	Na	Na	1%	10%	<1%	Alive	No
19	M	6	86%	Na	69%	Na	Na	Na	35%	10%	<1%	Alive	No
20	M	3	65%	Na	63%	Na	5%	<1%	1%	20%	20%	Alive	No
21	M	13	78%	Na	68%	<1%	Na	Na	<1%	80%	<1%	Alive	No
22	M	10	55%	Na	52%	Na	Na	Na	1%	40%	<1%	Alive	No
23	F	28	Na	Na	Na	Na	Na	Na	<1%	<1%	<1%	Alive	Yes
24	M	23	71%	Na	52%	83%	90%	82%	40%	10%	<1%	Alive	No
25	F	12	73%	Na	75%	Na	Na	Na	<1%	0	<1%	Alive	No
26	M	10	87%	Na	82%	Na	Na	Na	<1%	40%	8%	Alive	No

* Blasts % of total nucleated cells in the bone marrow aspirate. $ Blasts % of the total encountered events in the flow cytometry.

**Fig.1:**
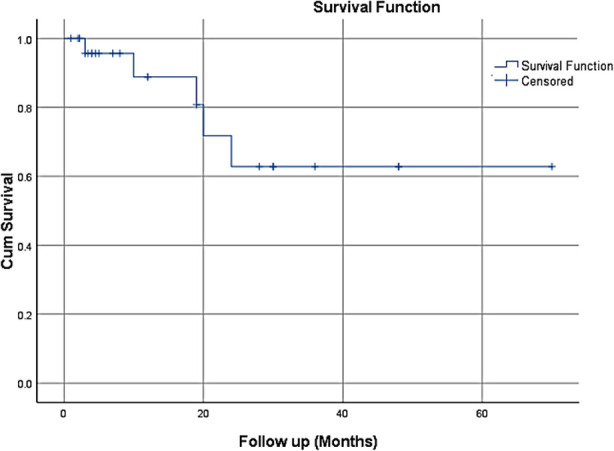
Kaplan-Meier survival plot showing probability of survival in relapsed T-ALL.

Relapse was recorded in five (19.23%) patients. All relapsed patients were over 10 years old, and two out of five (40%) had central nervous system involvement. Three (11.53%) patients did not survive during treatment, 2 of whom were under 10 years old. Overall, One out of five relapsed cases (20%) died within the study period. The documented causes of death included disease progression and multi-organ failure. In this study, the relapse rate for T-ALL was 19.23%, with an overall survival rate of about 64% (Fig.1), and the overall mortality rate was 11.53%.

## DISCUSSION

The present study aimed to investigate the clinical and laboratory characteristics, treatment outcomes, and survival rates of patients with T-lymphoblastic leukemia (T-ALL). The study examined 26 cases of T-lymphoblastic leukemia, predominantly in males with a mean age of 15.69±14.28 years. Immunophenotyping indicated high expression of CYTO-CD3, CD7, and CD5. Cytogenetic analysis revealed frequent translocations, notably TCR A/D and CDNK2A/CEP9. Although bone marrow examinations showed positive responses to therapy, relapse occurred in 19.23% of cases, particularly in patients over 10 years old with CNS involvement.

Acute lymphoblastic leukemia is considered the most common pediatric malignancy, constituting for about one third of all childhood cancers. Of which, ALL accounts for 75% of acute leukemia in this patient group.^5^ That is evident in the patients’ characteristics, as 55.4% of ALL cases are diagnosed at an age less than 20 years, 28% are diagnosed at 45 years or older and only 12.3% patients are diagnosed at 65 years or more. According to the origin of the malignant clone, arising either from B or T lymphoid progenitors, ALL is classified as B-ALL or T-ALL, respectively, the former entity accounting for 80-85% and the latter for 20-25% of ALL.^3^ The prognosis of T-ALL has been inferior compared to B-ALL in the past.

However, with more advances in therapeutic options, event-free survival (EFS) rates have improved over the years and currently exceed 85% in many internationally conducted trials.^14^,^15^ However, in the current study, the mortality rate during treatment was 11.53%, primarily affecting patients under 10 years old. The overall survival rate was approximately 64%, highlighting the challenges in managing T-ALL, particularly in relapsed cases and those with CNS involvement. Local papers have published data on event-free survival (EFS) of pediatric patients with ALL. Al-Nasser et al conducted a local series evaluating EFS in 509 pediatric patients who were treated either with local protocols (316 patients in the first arm) or international protocols (193 patients in the second arm).

The study reported a five years EFS of 30.6% in the first arm and 64.2% in the second arm (P<0.001), indicating significantly improved outcomes with international protocols.^16^ A study conducted by Ahmad et al. demonstrated improved overall outcomes in the treatment of ALL.^17^ In a more recent local study conducted by Al-Sudairy et al, the characteristics and treatment outcomes of 594 pediatric patients diagnosed with ALL were evaluated. The study found that the overall survival (OS) rate for patients with T-ALL, which constituted 10.7% of the patients, was 71.8%.^18^ Furthermore, another recent local study conducted by Jastaniah et al demonstrated no clinically significant difference in OS and event-free survival (EFS) between B-ALL and T-ALL.^19^

T-ALL exhibits distinctive immunophenotypic features, and multiparameter flow cytometry (FCM) plays a crucial role in detecting lineage-specific markers such as cytoplasmic CD3 and other relevant markers, thus aiding in the diagnosis of this condition. Additionally, FCM can be instrumental in identifying early T-cell precursor ALL (ETP-ALL), where blasts show negativity for CD1a and CD8, while expressing one or more myeloid/stem cell markers.^20^,^21^ Identifying poor prognostic factors at the time of diagnosis is essential as this will influence the treatment plan. Some poor prognostic factors include: complex karyotype, lack of mutation in either NOTCH1 or FBXW7 genes, del (17p)^6^,^11^ and the presence of CNS disease at the time of diagnosis.^16^

One additional important determinant of prognosis of the disease is evaluation of the Minimal Residual Disease (MRD) after the induction and the consolidation cycles. It has been identified that T-ALL shows a different response kinetics than B-ALL; with slower evidence of disease regression achieved in T-ALL. Despite the favorable outcome when establishing MRD negativity by the end of induction cycle, and by knowing the slow kinetic response of this entity, T-ALL patients who don’t achieve MRD negativity post induction but proved negative at the end of consolidation have a very favorable outcome with conventional chemotherapy.^22^

As mentioned earlier, identifying the prognostic factors is vital before initiating the management plan, as chemotherapy is usually delivered in a risk-based approach, using multiagent chemotherapy regimens that vary in different centers and include dexamethasone, asparaginase, methotrexate and intrathecal therapy. Therapy is usually given over 2-3 years with a possible addition of Cranial Radiotherapy (CRT) for selected high-risk patients; patients with CNS involvement or those who show MRD positivity. Achieving MRD negativity at the end of consolidation is a very important prognostic tool and accordingly, treatment delivered after this point is usually driven by the MRD response.^19^ Newer agents are being studied as possible therapeutic options in clinical trials. Another therapeutic option is Stem Cell transplantation (SCT), that is usually preserved for high risk disease.^14^

After achieving remission, T-ALL has high incidence of relapse with an Overall Survival (OS) of 50-60% in adult patients.^5^ With the application of risk-adapted therapy and increased supportive care, the survival rate of ALL among children has increased from 57 to 92%, however in 20% of children, relapses can still occur^23^, which have also been associated with poor outcomes. The incidence of high-risk leukemia and relapse is higher in adults (40-50%)^24^ as compared to children. This is in part due to the higher prevalence of high-risk molecular aberrations in adults.

### Limitations

It includes a small sample size and the restriction to a single-center setting, potentially limiting the generalizability of the findings. Additionally, long-term follow-up warrant caution in interpreting the results and emphasize the need for future research to address these limitations.

## CONCLUSION

With recent advancement in chemotherapy, the outcomes of T-ALL has improved significantly. In our study, the relapse rate was around 19%, the mortality rate was 11.5 %. There was significant reduction in blast percentages, indicating a positive early response of bone marrow to combination chemotherapy. This supports the better outcome of this entity with the use of aggressive and more effective chemotherapy than previous reports. Further studies, ideally performed prospectively, will further improve our understanding of the disease characteristics in our patient population.

### Authors Contribution:

**MA and** **SMA:** Conceived, designed and did statistical analysis & editing of manuscript, is responsible for integrity of research.

**MA, SMA and AFA:** Did data collection and manuscript writing.

**MA:** Did review and final approval of manuscript.
